# The practice of participatory action research: Complicity, power and prestige in dialogue with the ‘racialised mad’

**DOI:** 10.1111/1467-9566.13517

**Published:** 2022-08-24

**Authors:** Maria Haarmans, James Nazroo, Dharmi Kapadia, Charlotte Maxwell, Sonja Osahan, Jennifer Edant, Jason Grant‐Rowles, Zahra Motala, James Rhodes

**Affiliations:** ^1^ Department of Sociology University of Manchester Manchester UK

**Keywords:** coproduction, ethnicity, mental health, participatory action research, power, psychosis, race, stigma

## Abstract

Mental health service users in the UK have become increasingly involved in research over the last 2 decades partly as a consequence of research governance. Ethnic minority service users, however, point to power imbalances stemming from marginalisation and discrimination creating barriers to knowledge co‐production (Kalathil, J. (2013). Hard to reach? Racialised groups and mental health service user involvement.). Heavily influenced by Freire’s liberatory education, participatory action research (PAR) repoliticises participation where those most affected by injustice are central in both producing knowledge about injustice and implementing solutions. Ethnic minority people with lived experience of ‘severe mental illness’ (‘the racialised mad’) were appointed as coresearchers to work with academic researchers on a qualitative study exploring ethnic inequalities in ‘severe mental illness’. Drawing on Foucault’s notion of power as relational, we focus on three key aspects of productive power: (1) relational engagement and reciprocity, (2) positioning coresearchers as authentic researchers and (3) adopting an ethic of care, to explore complicity and resistance in reproducing hierarchies of knowledge and power when attempting to create and sustain a PAR process for collective analysis, action and solidarity. We utilise retrospective and recorded reflections over the course of the project. Finally, we discuss the ethical and methodological implications for contemporary sociological research into health and illness.

## INTRODUCTION

An orientation to critical inquiry, participatory action research (PAR) is typically employed for research questions that explore inequalities, extreme injustice, stigmatisation and marginalisation of ‘communities under siege’ (Fine & Torre, 2019, p. 435). A distinctive feature of PAR is the repoliticising of participation where those most affected by injustice are central in producing knowledge about injustice and implementing solutions. Through democratising knowledge production in this way, PAR …openly seeks to uncover and change the forms and mechanisms of domination and power’ (Maguire, 2000, p. 16). The ‘community under siege’, this article focusses on is the ‘racialised mad’ (Rose & Kalathil, 2019). The term ‘racialised’ refers to ‘…the placement of actors in racial categories or races…[where] the selection of certain human traits to designate a racial group is always socially rather than biologically based’ (Bonilla‐Silva, 1997, p. 469). Further, a range of resources—material, psychological, social and political—are distributed through modes of racial differentiation and hierarchies within a ‘racialized social system’. Borrowing the term ‘mad’ from survivor/service‐user Mad Studies movements, we use ‘racialised mad’ here to underline knowledges subjugated through the privileging of White, Eurocentric notions of ‘reason’ and as a ‘radical counter‐discourse to biomedical psychiatry’ (Sweeney, 2016, p. 43).

In this article, we trace and interrogate our attempts to implement the praxis of PAR in the context of a qualitative study on ethnic inequalities in severe mental illness^1^ (used here to refer to a diagnosis of a psychotic disorder) where extreme injustice sits across interdependent dimensions of structural, interpersonal and institutional racism (Nazroo et al., 2020). All phases of the study, including this article, were co‐produced with racialised people, with lived experience of ‘severe mental illness’ to promote inclusive knowledge production consistent with PAR (Gaventa & Cornwall, 2008).

Utilising Foucault’s (1977, 1979) relational view of power and paying particular attention to (changing) positionalities and subjectivities throughout the research process, we explore the power dynamics, challenges and dilemmas within a PAR praxis as well as our complicity with and resistance to processes and structures that reproduce power relations. Our use of PAR was intended to transform power relations as much as possible through resisting conventional processes of knowledge production. We draw on retrospective and recorded reflections as well as audio recordings of team meetings collected over the course of the project, privileging coresearcher voices since their accounts are rarely surfaced. In the next section, we briefly describe the background of the qualitative study and its organisation within a PAR framework. We do not discuss study findings here. Rather the focus is our experience of attempting a PAR praxis and the complicities involved in undertaking this orientation to inquiry within an academic institution.

Ethnic inequalities in severe mental illness and their association with broader social disadvantage and care experiences have been documented for more than 5 decades in the UK. Members of some racialised groups are more likely than their White counterparts to receive a diagnosis of psychosis (Black Caribbean and Black African people are five times more likely) (Halvorsrud, Nazroo, et al., 2019), experience coercive pathways to care (e.g., police and criminal justice system and detention under the mental health act) and receive biomedical as opposed to talking therapies (Halvorsrud et al., 2018). Persistent ethnic inequalities in broader social and economic disadvantages, including employment, housing and educational achievement (Jivraj & Simpson, 2015), are associated with ethnic inequalities in the risk of diagnosis or pathways to care. Little is known, however, about processes underlying these inequalities, how they are experienced, how they shape people’s lives and interactions with mental health and other institutions. It is these issues that were the focus of our research, leading us to choose a PAR approach in order to understand how ethnic inequalities are patterned beyond the individual and promote social and systems change to address them.

Over the last 2 decades, mental health service users have become increasingly involved in research on a continuum of levels from consultation to user‐led to user‐controlled where service users have ownership of the research process (Faulkner & Layzell, 1999; Staddon, 2013). However, though ‘user involvement’ has been systematically embedded through research governance in the UK, its depoliticising framework distinguishes it from PAR. Service users from racialised groups that do get a seat at the table point to power imbalances stemming from marginalisation and discrimination creating barriers to collaborative knowledge production (Kalathil, 2013). Indeed, Rose and Kalathil (2019) argue current co‐production in mental health research, which fails to disrupt the dominant discourse of psychiatry entrenched in Eurocentric epistemologies of universality and ‘reason’, cannot lay claim to the democratisation of knowledge production.

By appointing and training ethnic minority people with lived experience of severe mental distress as coresearchers to work with us as part of the research team, we envisioned creating, what Torre (2009, p. 110) calls ‘…a “contact zone”, a messy social space where very differently situated people could work together across their own varying relationships to power and privilege’; one where lived experiences of the ‘racialised mad’ (Rose & Kalathil, 2019) were centred.

To capture lived experiences across the life course in the study, we adapted a qualitative depth interviewing method, the biographical narrative interpretive method (BNIM) (Wengraf, 2001), to explore the biographies of 22 ethnic minority women and men with lived experience of psychosis. We felt BNIM was particularly compatible with PAR as it creates a space where participants can tell their story in their own way, less constrained by systems of relevance of the researcher, who remains a silent listener while the participant narrates their life story. Participants identifying as Black Caribbean, Black African, Black British, South Asian and mixed race, ranging in age from 22 to 63, resembled identities and age range of the research team. The resemblance of coresearcher team members to participants was purposeful in terms of recruiting ethnic minority coresearchers from these groups. We considered that shared lived experiences of mental health systems, severe mental distress and racialised and gendered identities would sensitise them to experiences of participants, enhancing the analysis consistent with a PAR praxis. In the next section, we present a critical overview of some of the ways power is conceptualised in PAR.

### Conceptualisations of power/knowledge in PAR

With historical roots in the global south (Freire, 1997; Kindon et al., 2007), theoretical traditions of critical race theory (Delgado & Stefancic, 2001) and feminist inquiry (Maguire, 2000), PAR is explicitly politicised, rejecting modernist claims to objectivity and value‐free theorising. With its origins in emancipatory education (most notably Freire’s work, 1997) and influenced by Gramscian notions of hegemony, the control of knowledge or ideologic manipulation to influence consciousness is understood to be critical to power in PAR (Gaventa & Cornwall, 2008). In these conceptualisations, the focus is on how power operates through knowledge production processes such as socialisation, education and the media creating monopolies of knowledge to shape consciousness. Internalised oppression operates to exclude people from awareness of power relations creating a ‘culture of silence’ of the oppressed.

Lived experience is considered a legitimate source of knowledge, with the recognition of a multiplicity of ways of knowing. One of PAR’s core methods, moving from personal experiences to social theorising (from the micro‐ to macro‐level of analysis) through the process of problem posing, continues as dialogue and reflection over time, each phase taking researchers to a deeper and more critical understanding of reality. Freire (1997) called this conscientisation where:

… people develop their power to perceive critically the way they exist in the world…they come to see the world not as a static reality, but as a reality in process, in transformation…the form of action they adopt is to a large extent a function of how they perceive themselves in the world.

(p. 83)

Foucault (1979), similarly understood subjectivity as dynamic, non‐unitary, context‐dependent and an effect of power/knowledge. He conceived of power as relational where ‘…power works through discourses, institutions and practices that are productive of power effects, framing the boundaries of possibility that govern action’ (Gaventa & Cornwall, 2008, p. 175). Foucault’s notion of power was not only negative but opened up possibilities for action through resisting boundaries. While he saw power as being enacted through practices as opposed to a commodity possessed, both he and Freire emphasised the possibility of transformation regarding how people see themselves in relation to their world, creating new capacities for change: what can be felt, thought or acted upon. By linking the creation of knowledge with social justice, PAR ultimately aims at three types of change (Maguire, 2000):

1. Development of the critical consciousness of both academic researcher and coresearcher;
2. Improvement of the lives of those involved in the research process;
3. Transformation of fundamental practices or power structures.

PAR researchers have, however, problematised the disconnect between theory and praxis (Cahill, 2007; Cornwall & Jewkes, 1995). Coresearchers rarely have complete control over the research with some not even wanting control. Involvement in choosing methods can be compromised due to lack of confidence and/or research knowledge thus potentially reinforcing social hierarchies. Furthermore, scientific rules dictate the validity of what issues and ‘methods’ for inquiry are deemed legitimate leading to inequalities in which groups get research funding as well as complicity with dominant knowledge production processes by adopting the language, methods and ‘rules of the game’.

Not often discussed in PAR literature, complexity of moral responsibility in asymmetrical power relationships presents inherent ethical challenges to relational aspects of wellbeing (Groot et al., 2019). Groot et al. (2019) identify Tronto’s (2013) ‘second‐generation ethics of care’ framework as useful for responding to these challenges. Typically gendered, emotional labour, ‘caring about’ (e.g., attentiveness to coresearcher needs), is an important part of a deliberate praxis of relational engagement and one that often remains invisible within conventional research processes, particularly in academic contexts. However, Groot et al.’s (2019) experience illuminates the dialectic between empowerment and disempowerment and between balancing awareness of vulnerabilities and power.

These dilemmas highlight the messiness in PAR praxis and the importance of researcher reflexivity of the complexity and contingency of power relations within a contemporary higher education environment.

## THE PRAXIS OF PAR: COLLECTIVITY AND COMPLICITY^2^

In this section, we critically reflect on our PAR praxis in the context of carrying out a health‐related qualitative study on ethnic inequalities in severe mental illness. We pay particular attention to our complicity with the institutional and power structures in which we are embedded. In doing so, we focus on team meetings as a site for ‘micro‐practices of power’ (Gaventa & Cornwall, 2008, p. 175): settings where our interactions had the potential to draw upon and reproduce existing power structures but also subvert and reconfigure them consistent with Foucault’s notion of power as relational. Team meetings provided a space for training, designing recruitment strategies, developing and refining data collection instruments, reflecting on experiences and issues of BNIM interviewing, conducting joint analysis and planning research dissemination. Following intensive training, nine team meetings and seven joint analysis meetings were held between July 2019 and August 2020. We discuss three productive effects of relational power that we attempted to mobilise to resist complicity with power structures and sustain solidarity: (1) a praxis of relational engagement and reciprocity, (2) positioning coresearchers as authentic researchers and (3) adopting an ethic of care. We structure our discussion of these themes temporally into the following sections: (1) Before data collection, (2) during data collection and (3) after data collection.

### Before data collection: Creating a praxis of relational engagement and reciprocity

As our focus of the study was the influence of racism on mental health and interactions with institutions, particularly the mental health system, we advertised positions for research associates at the University through a job posting calling for ethnic minority people with lived experience of severe mental illness. We appointed five coresearchers on casual contracts for 18 months to work with four academics with expertise in race, ethnic and gender inequalities: a postdoctoral research associate on a fixed‐term contract, with an interdisciplinary background in clinical and community psychology who was project lead; two lecturers in sociology; and a professor of sociology, who managed the research programme.

Emphasising the coresearchers’ combination of lived‐experience expertise, education and training, to our Human Resources partner allowed us to appoint them as ‘research associates’, normally requiring a PhD‐level qualification. In spite of these appointments disrupting academic professionalisation norms and being at odds with institutional expectations and infrastructures, this was a relatively smooth process perhaps because these posts were externally funded, project‐specific and short‐term.

Our research team (coresearchers and academics) included women and men who identify across a range of ethnic groups with diverse social locations and ages spanning 21–60. Coresearchers’ academic backgrounds included current students (undergraduate and postgraduate) and those with undergraduate and postgraduate qualifications. Although our social locations, disciplines and positioning within academia vary, we all share a passion for eliminating racial injustice in mental health services and beyond and a commitment to social change.

The first 5 days of our work together began with intensive training. A distinct and fundamental component of PAR, emphasised by Maguire (2000), is ‘…a commitment then to “be‐in‐relationship”’ (p. xvi). Thus, from the start we attempted to set a precedent for less hierarchical interactions and a deliberate praxis of relational engagement and reciprocity, where academics and coresearchers shared personal information more so than they would in other work settings. For example, we split into pairs telling each other about ourselves and sharing what we learnt about our partner to the larger group. In addition, as a sort of ‘biographical’ ice‐breaker (borrowed from Wengraf, 2019), each of us pointed to places on a world map explaining their significance in our lives.

Initially the idea of sharing such personal experiences about ourselves was uncomfortable for some academics who, socialised into conventional academic practices, were not used to working in this way. In fact, the project lead (with her experience in group facilitation based on feminist approaches) had to persuade the project manager of the usefulness of this approach. Afterwards, he acknowledged the benefit and we all noticed how these activities began to break down boundaries between the personal and professional, unsettling some of the assumptions we had about each other: ‘*A pleasant surprise was colleagues (bosses)*, *how honest they came across and how welcoming and supportive*. *I feel they went out of their way to make us feel like equals*. *Which is incredible and rare in my working experience… I felt it helps to create or deepen connections*…’[Coresearcher reflection].

Use of the term ‘*bosses*’ in parenthesis following ‘*colleagues*’ indicates that while this coresearcher experienced the academic facilitators as egalitarian colleagues, she remained conscious of the hierarchical dimensions of the working relationship.

A critical, though rarely discussed, feature of PAR praxis is the importance of group facilitation/process skills, such as ’drawing out the strengths of others’ (Grant et al., 2008, p. 597) enabling the disruption of dominant power relations and the centring of marginalised voices. The academics were surprised to discover how wholeheartedly coresearchers used this space to express their ideas, feelings and experiences, once a precedent had been set for positioning their knowledge as legitimate. In fact, the academics had to use their facilitation skills to keep to time rather than to draw out expression, a dynamic that continued throughout the research. Coresearcher reflections demonstrate how this structuring shaped power and group dynamics:

I appreciated their training delivery style, which was very much non‐hierarchical and very inclusive of the entire group… they were truly interested in hearing personal experiences and how these could contribute to the research project.

… I really enjoyed working with the rest of the team and felt very comfortable and able to speak even though I don’t have as much experience as everyone else… we were able to discuss collaboratively and disagree and debate without arguing.

How quickly the group started to form. Has this been achieved through having lived experience, being likeminded and or having a common goal?? And what else helped this process?

The last reflection in particular underlines a sense of solidarity arising from a mutual commitment to reducing ethnic inequalities in severe mental illness. Indeed, part of the training involved explication of our roles and expectations for our work including explicit acknowledgement of our power differentials. Academic researchers were transparent about the institutional processes that constrained opportunities for PAR and which underscored the dichotomous positions of ‘academic’ and ‘coresearcher’. This included the presence of strongly hierarchical lines of authority and command over resources (including budgets) and decision‐making; the explicit demand for the production of the highest quality academic publications; and no opportunities to offer secure employment contracts to coresearchers (with employment processes pushing for the equivalent of zero‐hour contracts).

Academic team members were open about their lack of previous experience of PAR; that we were all ‘learning by doing’ consistent with PAR (Maguire, 2000, p. 115). Included in this discussion was a coresearcher’s suggestion to jointly create a ‘living group agreement’ delineating how we wished to work together as a team. This not only foregrounded the importance of a praxis of relational engagement but introduced the academics to a new way of working together, not typically seen in conventional research teams.

While the broader area of inquiry, ethnic inequalities in severe mental illness, was already identified, and the BNIM approach established as our research method, part of the training involved collaboratively refining the research questions and materials. Throughout the research, coresearchers mobilised knowledge of being minoritised and stigmatised as a mental health service user, Black single mother, receiver of benefits, foodbank user and disabled person. They disagreed, debated, made suggestions and took the initiative with research activities. For example, during the review of study documentation, coresearchers advised on ways to make the adverts/participant information sheets accessible (e.g., large‐print, concise simplified versions) for older adults, those with a learning disability, various levels of literacy and speakers of other languages. Coresearchers also revised the participant debrief sheet, originally created by academic team members, producing an expanded flyer listing 60 low‐cost and/or free mental health services, including service user‐ and ethnic minority‐led organisations. As an example of the fluidity and multiplicity of subject positions (Cahill, 2007), here the notion of legitimacy was turned on its head, and academics felt unsettled by the visibility of their relative privilege.

As part of the BNIM training, facilitated by the project lead, academics and coresearchers became co‐learners, again reconfiguring the relations of power. Interviewing each other, we discovered together the power of this method and its significance for people who have experienced oppression and marginalisation. This type of experiential practice and dialogue further opened up the possibility for reciprocity, which led to adapting BNIM. For example, coresearchers identified the use of the term ‘*incident*’ (a cue word used in the BNIM second phase of interviewing for seeking detailed narrative), as problematic explaining that the use of this term is potentially emotionally triggering for people of colour due to coercive experiences with police, experiences that are often framed in official language as an ‘incident’. We subsequently dropped this term.

These processes illustrate mutuality of learning, recognition and deployment of the different types of knowledge typical of PAR, which both legitimised coresearchers’ expertise and greatly enhanced our original proposals in these areas. Throughout our work together, we intentionally created extensive space for dialogue with a minimum of didactic teaching, thereby opening up possibilities for PAR’s focus on capacity‐building and reciprocal learning (Freire, 1997).

However, team meetings followed a traditional academic format for the most part, with the project lead as chair, developing agendas in consultation with the project manager and circulating to the team for input. In establishing this approach, and throughout its application, we grappled with two particular issues. How far was positioning the project lead as ‘chair’ reproducing a hierarchical, masculinist academic practice antithetical to a PAR praxis? Was not a White woman in this position replicating the pervasive normativity of Whiteness within the Academy (Arday, 2018)? Why not rotate, or even dispense with, the role of chair? And wasn’t this also the case in relation to how the meeting agenda was established? Although this led to a tension between the desire to reduce power relations and the need to progress the research agenda within the time constraints, ultimately, pragmatics won. Moreover, due to the casual nature of coresearcher positions, proximity of academic researchers enabled them to meet more frequently to engage in research activity (e.g., develop meeting agendas), reinforcing the bifurcation of academic and coresearcher positioning. The project lead struggled with this tension, for increasing communication (to enable increased collaboration) meant in effect, a certain amount of unpaid work. Particularly relevant was that institutional structures—including funding requirements—dictated the need to deliver project outcomes within a particular timeframe. Relatedly, pressures on academic team members, in particular, to conform to accepted processes of CV building in order to progress their careers; and complex, inflexible, alienating, time‐consuming, though standardised, administrative processes, related to the ‘prestige economy of the academy’ (Leathwood & Read, 2013), surrounded each of our activities.

These institutional processes are, of course, expected, but they impact most significantly, and negatively, on those in more marginalised locations. In interactions with university systems, we were confronted, in a concrete way, with the embedded social and economic inequalities connected to racialised and stigmatised identities. These led to frustration, stress and feelings of powerlessness, particularly for the coresearchers. Academics deliberately used their positions within the university, drawing on their social and cultural capital, to persuasively advocate and mitigate some of the barriers faced by coresearchers. This included negotiating posting pay slips for coresearchers who needed hard copies to obtain disability benefits; providing emergency payments of salary where deadlines for submitting or processing claims were missed; and writing a letter to a governmental public service department when delays to a job start, despite already having received a job offer, created issues with the receipt of benefits.

Other institutional systems, we all had to navigate, that replicated power relations became apparent when formal appointment of coresearchers and data collection were delayed due to protracted processes for getting Disclosure and Barring Check (DBS, an official record stating criminal convictions) approval for all researchers. Difficulties in these processes directly reflected the life histories of coresearchers where experiences of a range of adverse circumstances, severe mental illness and racism made obtaining a DBS lengthy. For example, having a series of short‐term addresses considerably delays the DBS process because a search is required for each address. The complexity and length of this process, which academics had not anticipated due to their relative privilege, engendered anger towards them for not providing more support and a sense of powerlessness within the academic team for not being able to do more. Explaining what was happening behind the scenes (such as chasing up colleagues, dealing with numerous phone calls and emails), academics were transparent about their inability to do anything further. Nevertheless, the situation meant significant delays leaving a coresearcher unable to participate in data collection until later in the fieldwork period, reproducing her experiences of exclusion and marginalisation.

Prior to the development of the PAR work, there were extensive discussions within the broader research team on the terms we would use to describe the focus of our research, with us settling on ‘ethnic minority people’ and ‘severe mental illness’ (see Synergi, 2018, 2019). This terminology led to some disagreement and discussion among the PAR team. Some coresearchers were critical of the use of the word ‘minority’, which presumes a Eurocentric frame of reference. We discussed this decision at some length identifying various alternatives (e.g., specifying identities), none of which seemed adequate. In the end, we decided to retain the existing terminology with the rationale that it was already established and in use even though some coresearchers remained uncomfortable with the term. Additionally, some coresearchers preferred using diagnostic categories such as ‘schizophrenia’ in order to precisely define ‘severe mental illness’ in our research materials as opposed to specifying experiences of distress (e.g., hearing voices and paranoia), an approach consistent with the lived experience literature (Romme et al., 2009). Coresearchers contended that not using the diagnosis would exclude some people not presenting with these experiences. Academic team members, in particular, problematised the use of biomedical diagnostic categories as hegemonic discourses that could be argued to pathologise distress and oppress mental health service users. Such problem posing was intended as promoting conscientisation albeit drawing on well‐rehearsed debates that mobilised broader critical theory and associated research. However, while collaboration involves sharing knowledges and there was value in the open discussion and debate, perhaps more time for dialogue or involving coresearchers in drafting the research funding bid might have more straightforwardly engendered consensus. It is clear that power relations came into play in both instances, highlighting the importance of institutional constraints and academic legitimacy as a resource that reflected micro‐practices of power. A key challenge in making truly collaborative decisions is that marginalised groups often internalise academic hierarchies (Grant et al., 2008) consequently lacking a sense of legitimacy ‘…and confidence to challenge the guidance of “experts”’ (Cornwall & Jewkes, 1995, p. 1674). While coresearchers did in these instances challenge and refine decisions previously made by academics, internalisation of hierarchies (often unconscious), in both coresearchers and academics, may have prevented continued challenges or created space for further dialogue and mutual learning. Indeed, such internalisation is not limited to coresearchers. It is also, of course, present among the broader research team influencing how researchers interact with one another despite attempts to destabilise power relations. For example, the project lead (an early career female researcher) felt uncomfortable about the hierarchical meeting format positioning her as ‘chair’ for reasons cited above but was initially not confident enough to challenge the project manager:

I became aware that I was feeling intimidated by the project manager’s much more senior and scholarly experience in academia and in leading research teams. My anxiety makes it difficult to think ‘on my feet’ and articulate my thoughts clearly. Is this what some of the coresearchers experience?…

One could argue that academic researchers’ legitimacy is made indisputable through the mobilisation and articulation of theories and constructs that become internalised through working in academia, titles accompanying seniority and associated confidence and prestige. This reflects Foucault’s (1977/2000) elaboration of ‘…the system of differentiations that permits one to act upon the actions of others…’ (p. 344), so that status and academic legitimacy based on linguistic, cultural or competence differences are reified. Indeed, Maguire (2000) has pointed out how coresearchers ‘…may intuitively understand the concepts very well but lack the terminology that confers power’ (p. 2). A coresearcher’s reflections underline the impact of such differentiations when describing her negative experiences within learning institutions:

I find it very difficult in mainstream learning environments. I often cannot learn and express myself in the way that is expected of me…

### During data collection: Adopting an ethic of care

Attempting to adopt an ethic of care consistent with PAR and feminist methods, we started each meeting with a check‐in providing a space for sharing the personal. Check‐ins were often used as a vehicle to vent and express the frustration and anger experienced by coresearchers about inflexible, alienating bureaucratic university processes (e.g., issues with payment and delays in acquiring special equipment) related to the commercialisation and new managerialism of the UK academy (Leathwood & Read, 2013). Identifying with study participants, some also used this space to express anger and frustration with side effects of psychiatric medications, the sense of powerlessness in interactions with psychiatrists, and lack of access to therapy. Coresearchers’ lived experiences, therefore, were at the centre of these check‐ins.

Kiefer (1984) identified anger as a first step to empowerment, and, as Bell hooks has argued, in order to liberate subjectivity, ‘*we need to claim our rage*’ (hooks, 1995, p. 16). However, these eruptions became overwhelming for all of us, because they were difficult to contain and solutions were not immediately apparent, such that discussions became long and seemingly unproductive. In fact, many of us held on to meeting content for days afterwards and a coresearcher described how these expressions of anger were reminiscent of volatile family interactions. The meeting chair struggled with how to respond to these dynamics without shutting down discussion:

Why is facilitating so hard for me in this space? I’ve facilitated all types of challenging groups. Will intervening when others talk over each other, make me, with my white privilege, complicit through silencing and delegitimising anger? Reading hooks (1995) writing about the position of “the colonizer” who does not want to see “…the assertion of subjectivity…that surfaces when the colonized express rage”

(p. 12) resonates.

Some coresearchers reported that the space no longer felt safe, a side effect of allowing space for emotional outbursts and expressions of anger with one coresearcher feeling ‘silenced and excluded’, surfacing historical experiences of invalidation.

hooks (1995) also suggested we must engage with rage constructively, so it does not consume us. In a team meeting, we discussed the significant distress experienced collectively from the check‐ins and decided to experiment with alternatives, for example, reviewing our group agreement at the start of each meeting and using a ‘talking stick’ (an instrument of democracy in First Nations and many indigenous cultures in which only the person holding the stick speaks). However, none seemed effective: coresearchers continued to talk over one another and check‐ins dominated the meeting space. We therefore decided to dispense with check‐ins and instead utilise lived experience knowledge in other spaces (e.g., reflections about the experience of interviewing and joint co‐analysis sessions) as well as reserving an explicit space for the sharing of personal experiences that were not project‐related in the final section of the agenda. Most coresearchers expressed relief about this solution, with one reflecting: ‘*eradication of the* “*check‐in*”, *clears the way for just getting on with actual work… [e]ven though the original check‐in was well intended*’.

This experience underlines the importance of being attentive to the interpersonal dynamics within the team as well as the structural inequalities faced daily by coresearchers; of being mindful of how we hold power in a space, of how inaction, not using one’s power, can be a form of tacit complicity with structural power relations. Attempts to promote power‐sharing through the chair (and other academics) staying silent and not steering discussion may have inadvertently reinforced micro‐practices of power.

In addition to team meetings, the project lead provided one‐hour debriefing sessions immediately following each interview, individual coaching, mentoring and informal catch‐ups in a manner consistent with an ethic of care (Groot et al., 2019; Ward & Gahagan, 2010).These catch‐ups became especially important when team meetings became infrequent and during the COVID‐19 lockdown, because they sustained engagement and communication. They were valued by the project lead and coresearchers for maintaining connection and mutual affection.

We found that micro‐practices of power can extend to mutuality in ‘caregiving’ where relations of power are reconfigured through reciprocity (Chesnay, 2016). Surprisingly, the notion of ‘caregiving’ as bidirectional and mutual (Groot et al., 2019), drawn from Tronto’s (2013) ‘second‐generation ethics of care’, is not often written about in PAR studies though it was apparent in our interactions. This is reflected in the email quoted below, where a coresearcher provided support to the project lead following a particularly emotional meeting.

Hi [*Project Lead name*],

I’m just checking in to see how you are today, so how are you?

Yesterday was quite emotional with many feelings around the table. I also wanted to say thank you for crying! Not because it makes you human, I knew that from when I first met you, but because you were able to cry with/in front of us…

I send you big hugs,

Mutual care‐giving illustrates the potential for ‘being‐in‐relationship’ and for human connection in participatory work where emotional engagement can disrupt power differentials by deploying alternative subjectivities as valued and respected colleagues (Maguire, 2000).

### After data collection: Positioning coresearchers as authentic researchers

In this section, we examine the content and process of our joint analysis sessions paying particular attention to power, representation and agency of team members (Cornwall & Jewkes, 1995; Foucault, 1979; Freire, 1997). Not only did coresearchers conduct and transcribe the majority of BNIM interviews (15 out of 22), they also analysed the data both individually and jointly. While all team members attended one or more meetings, not everyone attended all seven. For five of these meetings, we individually analysed selected interview transcripts and recorded our analyses on a document prior to the meeting, which were later compiled, shared and discussed at the joint meeting.

During analysis, the risk exists that coresearchers’ lived experience and local knowledge will be subjugated and academic researchers’ interpretations privileged (Grant et al., 2008). Therefore, academic team members decided to centre the voices and analyses of coresearchers expecting that this would draw upon their subjectivities and lived experience; a deliberate strategy to both disrupt power differentials and maximise the limited time we had available to work jointly. Increasingly the coresearchers contributed their interpretations of the data from a position of confidence, as suggested by the passion and enthusiasm with which they were delivered and illustrated by their reflections:

It’s almost like, I come alive, when we have these discussions’.

I personally became more confident that I had valuable information to contribute …

I think there’s something here about shared learning/knowledge distribution too – myself and another coresearcher have talked quite a bit about how we would take notes on each other’s reflections to go away and do some reading.

Three joint analysis meetings were recorded, transcribed and, alongside the independent analysis documents, thematically analysed by the project lead. This analysis was then discussed in a further joint analysis meeting and contributed to a framework for future analysis (Gale et al., 2013). These processes legitimised coresearcher contributions, helping to increase their confidence and sense of agency regarding the research process.

Unlike some participatory research where involvement is merely consultative, here, more than just taking part (Cornwall & Jewkes, 1995), it involves active interpretation and making choices about meaning that enhances analysis within a praxis of relational engagement and reciprocity. Coresearchers are being positioned, and are positioning themselves, as authentic researchers revealing the potential for (trans)formation of subjectivities despite the presence of an enduring operation of power in our encounters.

But what prevented us from challenging, or even subverting, the demands of the UK academy that reproduced power relations within our research team and demands that often hinder more critical or innovative research? Leathwood and Read (2013), drawing on Foucault’s concept of disciplinary technologies and self‐governance, point out how academics ’are both governed and self‐governing’ (p. 1164). They describe how we are conditioned to struggle to produce a stream of never‐ending research outputs and become entrepreneurial ’selling our work and ourselves in the prestige economy of the academy’ (p. 1165). Such processes produce new subjectivities that render rejection or challenge of these practices unimaginable particularly when also subject to precarity from ‘fixed term contracts’, early stage careers and minoritised identities. An academic’s reflections foreground the need to juggle extremely demanding workloads and manage the emotional labour involved in PAR:

Since the beginning of the new semester, I’ve been really busy and so I’ve not felt much inclination to write this reflective piece because I’m tired from work and maybe my ability to reflect is not as good as it is usually.… I think it’s a very emotionally challenging project …and at times, I have wondered about my own ability to cope with this project and its contents, as well as other roles that I have for teaching, supervision, academic advising etc. …So Monday and Tuesday, I have felt like I have been listening to people’s problems, worries, and things about their difficult lives a lot or I’ve been lecturing/advising/mentoring…

Such pressures and sheer exhaustion lead to a degree of complicity with academic power structures, as we, both academics and coresearchers, feel the need to ‘play the game’ and prove our worth and capabilities through producing ‘acceptable’ outputs (including this article) due to threats of precarity in our jobs. By not engaging in sustained overt protest or resistance to these structures, are we not being socialised into ‘ideal neoliberal subjects’ (p. 1164)?

## DISCUSSION

In this article, we interrogate the power dynamics (structural and relational) at play within our PAR praxis, in what is, to our knowledge, the first PAR project conducted jointly by ethnic minority people with lived experience of ‘severe mental illness’ and academic researchers. To do this, we draw on a Foucauldian perspective of power as relational and productive. Though we attempt a critically reflexive analysis consistent with PAR, due to our particular standpoints, we may not be conscious of all of the ways in which we replicated or reified those power relations that have become normalised and naturalised within academia and beyond (Foucault, 1979).

Fundamentally, we found that an intentional praxis of relational engagement and reciprocity, typical of PAR, was crucial to establishing a bond (Fine & Torre, 2019; Maguire, 2000). Our willingness to be open about our values, passions and standpoints; to share personal experiences (initially causing discomfort for some academics) and to be vulnerable and show and accept each other ‘warts and all’ (Maguire, 2000) helped earn trust across our team. While knowledge, expertise and care of each member of the team was legitimised and valued, ultimately the research, situated within the university, was controlled by the academic researchers.

Adopting Freire’s (1997) dialogical ‘problem posing’ method, while not without its challenges, created a collective space for mutual learning, sharing of lived experiences and conscientisation. Conscientisation, consistent with PAR, is the recognition of a multiplicity of ways of knowing and privileging the deep knowledge arising from oppression. Coresearchers, sensitised by their own lived experiences of racialisation and stigmatisation, drew on experiences of being prescribed powerful antipsychotic medications, being an in‐patient, living in poverty, receiving a psychiatric diagnosis, experiencing racism and of encounters within academia, throughout the research to understand and interpret the data that was mutually produced. Generating knowledge intersubjectively through dialogue, collective processes (Kindon et al., 2007) and ‘productive complicities’ in this way can lead to the creation of new subjectivities, even though this process was at times painful.

An overarching finding from analysis of our PAR praxis was that mutuality and reciprocity as micro‐practices of power went some way to positioning the coresearchers as authentic and legitimate researchers and connected members of the research team.

We found that micro‐practices of power can extend to mutuality in ‘caregiving’. An ethic of care that is bidirectional was an integral part of our PAR praxis opening up possibilities for reconfiguring power relations and subjectivities. This attunement to one another’s emotions and needs is something that does not often appear in the PAR literature (Groot et al., 2019). Attention to an ethic of care is not meant to displace focus from the social–structural oppressions that create distress or to further structures of individualisation (Burman, 2006), but it was important for sustaining our collective solidarity in the context of such oppression.

Coresearchers’ changing subjectivities were evident in terms of increased capabilities and awareness of what is possible (Foucault, 1979; Freire, 1997). For example, a coresearcher who decided to apply (and has since been accepted) to a clinical doctorate programme attributes this decision to the PAR experience that shifted belief in her capability; a poster presentation for the British Psychological Society won the judges’ prize; and coresearchers contributed blogs and podcasts.

However, these practices were constrained by institutional power structures fundamental to the UK academy within which we operated. While we were able to mobilise our status and positions within the university to mitigate some harms, we were consciously complicit with institutional forces. Like many other PAR projects within a university context (Dewa et al., 2021; Dillon, 2014), the need to be pragmatic limited more aspirational elements of our PAR. For example, as Dewa et al. (2021) found in their study, co‐produced with young people with mental health difficulties, interactions, tasks and unexpected delays or events often result in more time and funds needed than anticipated, despite best efforts to plan for these. We did not anticipate the time needed for our dialogic practice and for adapting circumstances to enable inclusion of all coresearchers. We discovered that enabling participation meant flexibility for dipping in and out of project activities due to individual preferences, skills and personal circumstances. This is consistent with Halvorsrud et al.’s (2019a) meta‐analysis on co‐production in health care that concluded flexibility, accommodation to circumstances and varied needs were crucial elements of participatory processes.

### A way forward: Ethical and methodological implications for medical sociology

It is important to acknowledge openly that inequalities within the research relationship can only be partially mitigated through PAR processes. There remains a need to attend closely to the complexity of power and interpersonal dynamics at play (Grant et al., 2008). Primary constraints to the PAR praxis were related to the institutional power structures we were operating within; and an essential consideration for medical sociologists interested in critical inquiry and emancipatory methodologies. In doing such work, it is an ethical responsibility for medical sociologists to engage with power and privilege prior to initiating PAR, anticipating and reflecting on the implications of this kind of work for coresearchers’ lives. While coresearchers were involved in all stages of the research process, involving them at the stage of drafting the research bid would have enabled a different approach, and likely, a different outcome, to the focus and design of the study. This could have further, and perhaps more fundamentally, both revealed and subverted power differentials in the research process. Such early involvement may also have mitigated some of the issues confronting coresearchers that the academics, lacking lived experience, did not anticipate. However, the uncertainty around project funding might also have amplified inequalities across the team.

Much of the research agenda and activities were decided by academic team members (i.e., meeting structure and analysis session format) not unlike many studies in the health field incorporating PAR (Halvorsrud, Kucharska, et al., 2019). This was a consequence of the institutional constraints within which we were embedded and the fact that academic team members have comparatively more experience in these things; in fact, this is not dissimilar from how other projects with research associates are organised.

While PAR processes caused us to reflect on and to be conscious of our actions and their consequences for the coresearchers thereby helping us navigate our way through it in a way to maximise benefit and limit harm, such increased consciousness is only a first step. As a team, we experienced the power structures we are working within as robust to strategies we could use to challenge them, contributing to our complicity in sustaining them. If we are to engage in emancipatory methodologies that can transform oppressive practices and power structures, we need to, as Maguire (2000) has argued, ‘dig where you stand’ (p. xv) and push for broader social change both within and beyond the academy.

## AUTHOR CONTRIBUTIONS

**Maria Haarmans**: Conceptualisation (Lead); Data curation (Lead); Formal analysis (Lead); Investigation (Lead); Methodology (Lead); Project administration (Lead); Supervision (Lead); Writing—original draft (Lead); Writing—review & editing (Lead). **James Nazroo**: Conceptualisation (Lead); Data curation (Lead); Formal analysis (Equal); Funding acquisition (Lead); Investigation; Supporting, Methodology (Equal); Project administration (Supporting); Resources (Lead); Supervision (Lead); Writing—review & editing (Supporting). **Dharmi Kapadia**: Conceptualisation (Supporting); Formal analysis (Equal); Investigation (Supporting); Methodology (Supporting); Supervision (Supporting); Writing—review & editing (Supporting). **Charlotte Maxwell**: Conceptualisation (Supporting); Formal analysis (Equal); Investigation (Equal); Methodology (Supporting); Writing—review & editing (Supporting). **Sonja Osahan**: Conceptualisation (Supporting); Formal analysis (Equal); Investigation (Equal); Methodology (Supporting); Writing—review & editing (Supporting). **Jason Grant‐Rowles**: Conceptualisation (Supporting); Formal analysis (Equal), Investigation (Equal); Methodology (Supporting); Writing—review & editing (Supporting). **Jennifer Edant**: Conceptualisation (Supporting); Formal analysis (Equal); Investigation (Equal); Methodology (Supporting); Writing—review & editing (Supporting). **Zahra Motala**: Conceptualisation (Supporting); Formal analysis (Equal); Investigation (Equal); Methodology (Supporting); Writing—review & editing (Supporting). **James Rhodes**: Conceptualisation (Supporting); Formal analysis (Equal); Investigation (Supporting); Methodology (Supporting); Supervision (Supporting); Writing—review & editing (Supporting).

## Data Availability

The politically and personally sensitive nature of the data, which is qualitative, makes them highly disclosive, thus supporting data is not available.

## ENDNOTES

^1^
Throughout this article, we use the term ‘severe mental illness’ noting that this socially constructed categorisation is one of the primary ways in which mental health service users are othered and oppressed. We have written elsewhere extensively about our use of terminologies including participatory action research and ethnic minority people (Synergi, 2018, 2019).

^2^
We choose to limit the examples in this section due to risk of disclosure.
